# Death receptor 6 (DR6) antagonist antibody is neuroprotective in the mouse SOD1^G93A^ model of amyotrophic lateral sclerosis

**DOI:** 10.1038/cddis.2013.378

**Published:** 2013-10-10

**Authors:** G Huang, X Lee, Y Bian, Z Shao, G Sheng, R B Pepinsky, S Mi

**Affiliations:** 1 Biogen Idec Inc., 14 Cambridge Center, Cambridge, MA 02142, USA

**Keywords:** ALS, DR6, motor neuron, neurofilament, neurodegeneration, NMJ

## Abstract

Amyotrophic lateral sclerosis (ALS) is a neurodegenerative disease characterized by the death of motor neurons, axon degeneration, and denervation of neuromuscular junctions (NMJ). Here we show that death receptor 6 (DR6) levels are elevated in spinal cords from post-mortem samples of human ALS and from SOD1^G93A^ transgenic mice, and DR6 promotes motor neuron death through activation of the caspase 3 signaling pathway. Blocking DR6 with antagonist antibody 5D10 promotes motor neuron survival *in vitro* via activation of Akt phosphorylation and inhibition of the caspase 3 signaling pathway, after growth factor withdrawal, sodium arsenite treatment or co-culture with SOD1^G93A^ astrocytes. Treatment of SOD1^G93A^ mice at an asymptomatic stage starting on the age of 42 days with 5D10 protects NMJ from denervation, decreases gliosis, increases survival of motor neurons and CC1^+^ oligodendrocytes in spinal cord, decreases phosphorylated neurofilament heavy chain (pNfH) levels in serum, and promotes motor functional improvement assessed by increased grip strength. The combined data provide clear evidence for neuroprotective effects of 5D10. Blocking DR6 function represents a new approach for the treatment of neurodegenerative disorders involving motor neuron death and axon degeneration, such as ALS.

Amyotrophic lateral sclerosis (ALS) is an adult neurodegenerative disease characterized by the selective death of upper and lower motor neurons and axon degeneration leading to progressive paralysis.^1^ Disease onset typically occurs at an age of 50–60 with an incidence of 1 in 50 000 annually and a cumulative lifetime risk of 1 : 300.^2, 3^ Mean survival following diagnosis is only 18 months, although some individuals can live for a decade or more. Most ALS cases present with no clear familial history, ∼10% have a strong inheritance pattern and deemed familial ALS (FALS). Analysis of these pedigrees has enabled identification of disease causing gene polymorphisms, with the best characterized being mutations in copper/zinc superoxide dismutase 1 (SOD1) gene.^4^ Other disease-causing gene mutations have been found in the TDP-43,^5^ C9orf72,^6^ *FUS/TLS,*^7^ angiogenin^8^ and *VAPB*^9^ genes.

About 20% of FALS have a SOD1 mutation.^4^ The identification of SOD1 mutations in ALS has allowed the development of animal models of the disease to study molecular pathogenic events *in vivo* and to study drug survival effects and improvements of pathology. The most extensively used ALS model is the SOD1^G93A^ mouse, which expresses high levels of the human mutant protein under the control of the SOD1 promoter. Many studies claiming potential therapeutic agents, which extended survival in this model, such as Thalidomide,^10, 11^ Olesoxime,^12^ and Dexpramipexole^13^ failed in clinical trials. Nevertheless, the model provides a great tool to study motor neurons and axon degeneration progression by histology, because the SOD1^G93A^ mice develop a motor neuron disease with a pathology that recapitulates important aspects of ALS following the disease progression.^14, 15^ Both SOD1^G93A^ mice and ALS patients show significant synaptic degeneration, gliosis (astrocytic activation), caspase activation, motor neuron death and degeneration of neuromuscular junctions (NMJ).^16, 17, 18, 19^ Disease progression also leads to increased levels of phosphorylated neurofilament heavy chain (pNfH), a major structural component of motor neuron and axon, into cerebrospinal fluid and blood circulation, in both SOD1^G93A^ mice and ALS patients, which correlates with disease severity.^20, 21, 22^

DR6 belongs to the tumor necrosis factor receptor super family and contains the four highly conserved cysteine-rich extracellular domains implicated in ligand binding and oligomerization, and a cytoplasmic death domain that upon receptor oligomerization activates diverse downstream targets, including caspases.^23, 24^ There is increasing evidence that DR6 has an important role in neuronal cell death. DR6 has been reported to induce neuronal cell death and axon degeneration during central nervous system development by binding N-terminal beta-amyloid precursor protein (N-APP) through activation of the caspase signaling pathways,^25^ and by complexing with p75 neurotrophin receptor (p75^NTR^) responsible for *β*-amyloid (A*β*) inducing cytotoxicity of cortical neurons.^26^ DR6 also mediates oligodendrocyte cell death during development,^27^ and oligodendrocytes have been recently implicated to contribute to motor neuron disease in SOD1^G93A^ mice.^28, 29, 30^ Here we demonstrate that DR6 expression is upregulated in motor neuron of ALS post-mortem samples and SOD1^G93A^ mice. Blocking DR6 by an anti-DR6 antibody promotes motor neurons survival *in vitro* and *in vivo*. The neuronal protective effects mediated by anti-DR6 antibody can be detected as early as the age of 60 days in SOD1^G93A^ mice after 2 weeks of treatment, as evident by decreased denervated NMJ using histology assessment and decreased pNfH levels in serum. The study identifies DR6 as a novel therapeutic target for the treatment of neurodegenerative diseases involving motor neurons and axon degeneration.

## Results

### DR6 is upregulated in ALS post-mortem samples and SOD1 ^G93A^ mice spinal cords

**Figure 1:**
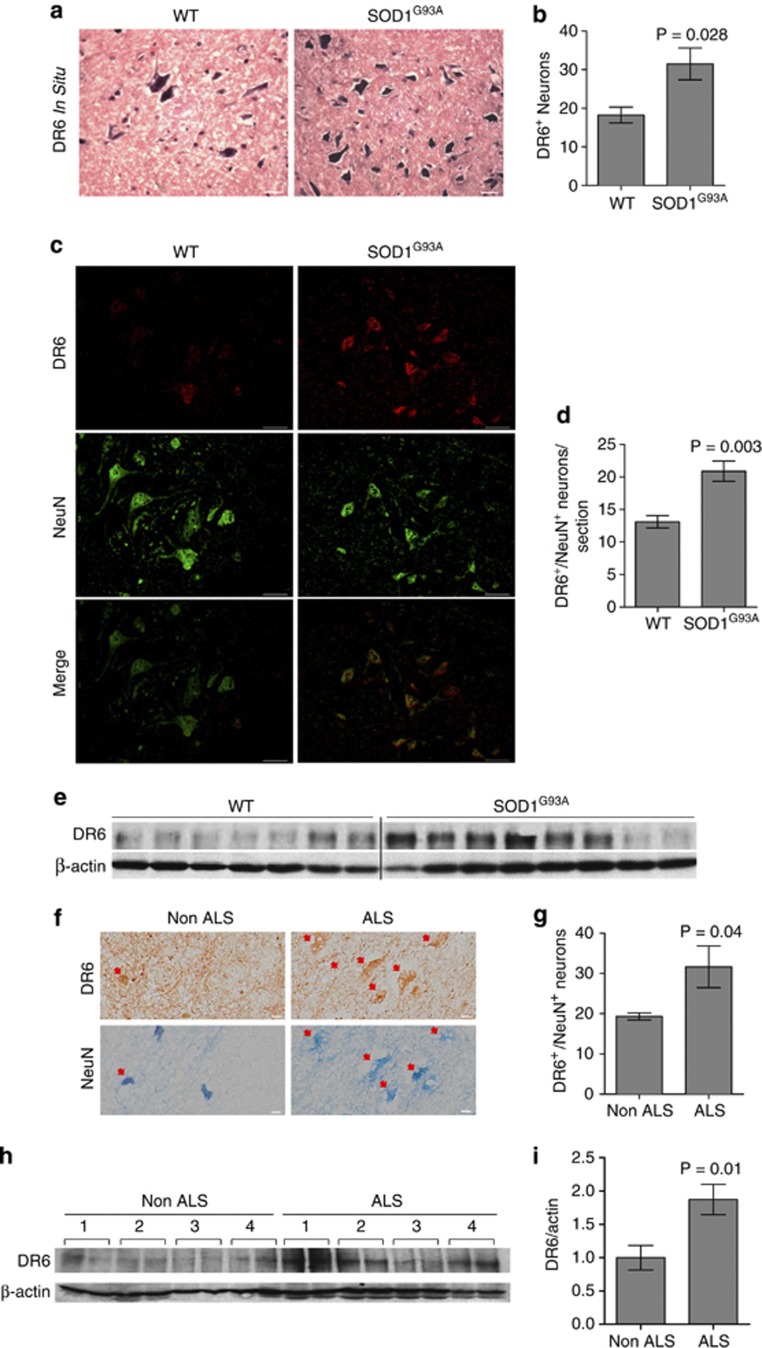
DR6 mRNA and protein levels are upregulated in spinal cord of SOD1^G93A^ mice and ALS post-mortem samples. (**a**) *In situ* hybridization of DR6^+^ motor neurons at the age of 60 days SOD1^G93A^ and WT mice, scale bar=25 *μ*m. (**b**) Quantification of DR6^+^ motor neuron number from **a**, *n*=4 fields/3 animals/group. (**c**) IHC analysis of DR6 expression in SOD1^G93A^ and WT mice lumbar spinal cord ventral horn region (age 60 days), DR6 (red), NeuN (green), scale bar=50 *μ*m. (**d**) Quantification of DR6^+^/NeuN^+^ (>20 *μ*m) neuron number from **c**, *n*=9 sections/3 animals/group. (**e**) Western blot analysis in SOD1^G93A^ and WT mice spinal cord (age 60 days) for DR6 expression, WT: *n*=7, SOD1^G93A^: *n*=8. *β*-actin was used as an internal control. (**f**) IHC analysis of DR6 expression in human non-ALS or ALS post-mortem spinal cord tissue, adjacent frozen sections were used to stain DR6 (brown, top panel) and NeuN (blue, bottom panel), respectively, arrows denote DR6^+^ (top panel) or NeuN^+^ (bottom panel) neurons, respectively, scale bar=20 *μ*m. (**g**) Quantification of DR6^+^/NeuN^+^ (>30 *μ*m) neuron number from **f**, *n*=3 samples/group. (**h**) Western blot analysis showing DR6 expression in human post-mortem spinal cord samples run in duplicates. *β*-actin was used as an internal control. (**i**) Quantification of DR6 expression from **h** plus an additional nine ALS and five non-ALS spinal cords by densitometry. Total ALS samples: *n*=13, total non-ALS samples: *n*=9. Data presented as the ratio of DR6 over actin. Data in **b**, **d**, **g**, and **i** were shown as mean±S.E.M. *P*-values were determined by two-tailed unpaired *t* test

DR6 is broadly expressed by developing neurons, including motor neurons, and has been linked to regulating neuronal survival during development.^25^ We investigated if DR6 was involved in ALS pathology by first determining if DR6 mRNA is increased in SOD1^G93A^ transgenic mice, an extensively characterized animal model for ALS. Ventral horn motor neurons in the lumbar spinal cord of presymptomatic SOD1^G93A^ mice were labeled for DR6 expression by *in situ* hybridization and counted. DR6 antisense RNA strongly stained motor neurons (Figure 1a). The number of DR6^+^ neurons was 1.7-fold higher in SOD1^G93A^ than in age-matched non-transgenic animals (Figure 1b). DR6^+^ SOD1^G93A^ neurons were smaller and stained more intensively than control (Figure 1a), suggesting that DR6 expression is upregulated in motor neurons to induce the pathological changes. To determine if DR6 protein levels are increased in spinal cords of SOD1^G93A^ mice, we performed immunohistochemistry (IHC) and western blot using anti-DR6 antibody, 6A12. There were 1.6-fold more DR6^+^/NeuN^+^ (>20 *μ*m) neurons in ventral horn of the spinal cord in SOD1^G93A^ compared with control at as early as the age of 60 days (Figures 1c and d). Western blot analysis showed an increase in DR6 protein levels in SOD1^G93A^ spinal cord (Figure 1e). The specificity of this antibody was confirmed using DR6-null (*Tnfrsf21*^*−/−*^) mice tissue (Supplementary Figure 1).

Next, we investigated whether DR6 expression was upregulated in human ALS post-mortem cervical spinal cord tissue by IHC and Western blot. A 1.6-fold increase in DR6^+^/NeuN^+^ (>30 *μ*m) neurons was observed in ALS samples compared with age-matched non-ALS controls by IHC (Figures 1f and g). Consistent with this finding, western blot analysis showed a twofold increase in DR6 protein levels in the ALS samples (Figures 1h and i). The presence of elevated DR6 mRNA and protein levels in spinal cord of SOD1^G93A^ mice and human ALS post-mortem samples suggests that increased DR6 levels may contribute to ALS pathology.

### Blocking DR6 promotes motor neuron survival *in vitro*

**Figure 2:**
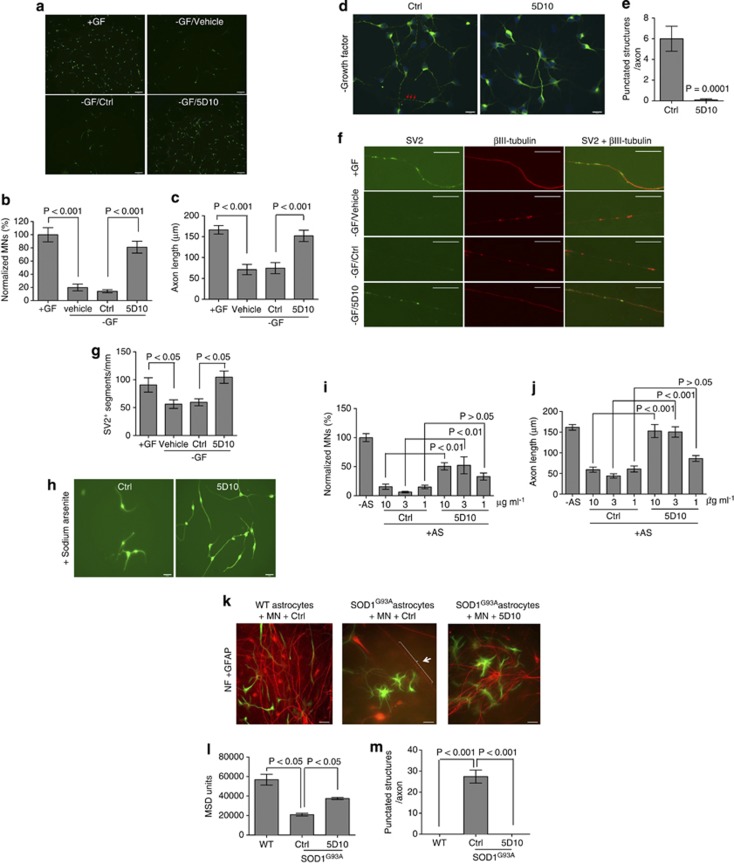
Anti-DR6 antibody promotes human motor neuron survival and preserves axon integrity *in vitro*. (**a**) ICC images of human motor neurons following growth factor removal, and treated with 10 *μ*g/ml isotype control antibody or 5D10, NF (green), scale bar=95 *μ*m. (**b**) Quantification of surviving motor neuron number from **a**, growth factor supplemented condition was used as a positive control (100%), *n*=12–48 fields/group. (**c**) Quantification of axon length from **a**, *n*=∼30 axons/15 fields/group. (**d**) ICC images of rat motor neurons treated with 10 *μ*g/ml control antibody or 5D10 following growth factor removal, MAP2 (green), arrow, punctated structures along axons, scale bar=15 *μ*m. (**e**) Quantification of axon punctated structures from **d**, *n*=10 axons/10 fields/group. (**f**) ICC images of rat motor neuron axons treated with 10 μg/ml control antibody or 5D10 following growth factor removal, SV2 (green), *βIII*-tubulin (red), scale bar=10 μm. (**g**) Quantification of SV2^+^ segmented structures per mm axon from **f**, *n*=10 axons/10 fields/group. (**h**) ICC images of human motor neurons following sodium arsenite, and treated with 10 *μ*g/ml 5D10 or control antibody, NF (green), scale bar=25 *μ*m. (**i**) Quantification of surviving motor neuron number from **h**, sodium arsenite minus condition was used as a positive control (100%), *n*=12 fields/group. (**j**) Quantification of axon length from **h**, *n*=∼24 axons/12 fields/group. (**k**) ICC images of rat motor neurons in co-cultures with purified astrocytes from WT or SOD1^G93A^ mice, and treated with 10 μg/ml 5D10 or control antibody. NF (red), GFAP (green), arrows denote axon punctated structures, scale bar=25 *μ*m. (**l**) Quantification of NF levels from **k** by MSD analysis, *n*=2 samples/group. (**m**) Quantification of axon (longer than 250 *μ*m) punctated structures from **k**, *n*=12 axons/12 fields/group. Data in **b**, **c**, **e**, **g**, **i**, **j**, **l**, and **m** were shown as mean±S.E.M. *P*-values in **e** were determined by two-tailed unpaired *t*-test, and in **b**, **c**, **g**, **i**, **j**, **l** and **m** by one-way analysis of variance (ANOVA followed by Bonferroni's test)

DR6 was previously reported to induce developmental neuronal cell death.^25^ Combined with our data that DR6 is upregulated in motor neurons of SOD1^G93A^ mice and human ALS post-mortem samples, we hypothesized that blocking DR6 could promote motor neuron survival. To test this hypothesis, we first determined whether DR6 is expressed in cultured human motor neurons. Immunocytochemistry analysis (ICC) of human motor neurons revealed that anti-DR6 antibody 6A12, but not control antibody, co-stained motor neurons visualized with anti-neurofilament (NF) antibody (Supplementary Figure 2). Staining occurred in both the cell body and axons. We next determined whether blocking DR6 by anti-DR6 antagonist monoclonal antibody 5D10, as described previously,^27^ protected motor neurons from death using three methods: growth factor removal, sodium arsenite to induce mitochondrial oxidative stress and astrocyte (SOD1^G93A^) induced cytotoxicity in motor neuron/astrocyte co-cultures.^31, 32^ Healthy axons were seen when human motor neurons were cultured for 24 h with growth factors as shown in Figure 2a. Growth factor removal led to a fivefold reduction in the number of surviving neurons (compared with growth factor-supplemented condition), whereas 5D10 treatment following growth factor removal increased the number of surviving neurons by fivefold (Figures 2a and b). Axons in anti-DR6 antibody-treated neurons were twofold longer than control antibody-treated cells (Figures 2a and c). Blocking DR6 by 5D10 also promoted rat motor neuron survival following growth factor removal (data not shown). In addition, there were many punctated structures along axons in control-treated neurons, indicating disruption of axon microtubule structure and degeneration;^33, 34^ these punctated structures were not detected in 5D10-treated neurons (Figures 2d and e). To further confirm 5D10 treatment protects axon integrity, axons were double-stained for synaptic vesicle protein 2 (SV2, a synaptic marker) and βIII-tubulin. In the presence of growth factor, cultured neurons contained a segmented SV2^+^ staining pattern characteristic of healthy axons and no *β*III-tubulin^+^ punctated structures (Figure 2f). Growth factor removal led to a 50% reduction in the number of SV2^+^ segmented structures (Figures 2f and g) and an increase in punctated βIII-tubulin^+^ axons. 5D10 treatment following growth factor removal led to a twofold increase in the number of SV2^+^ segmented structures versus control treatment (Figures 2f and g) with no detectable punctated βIII-tubulin^+^ axons. Similarly, 5D10 treatment led to a maximal threefold increase in motor neuron survival and axon length following sodium arsenate treatment at concentrations of ⩾3 *μ*g/ml (Figures 2h–j). Third, we tested anti-DR6 antibody effect on motor neuron protection from SOD1^G93A^ astrocyte-induced toxicity (Figures 2k–m). In this study, purified astrocytes from the brains of 2-month-old SOD1^G93A^ transgenic or control mice were co-cultured with purified rat motor neurons. Motor neurons and astrocytes were visualized by ICC using anti-NF and anti-GFAP antibody, respectively. The toxic effect of the SOD1^G93A^ astrocytes was clearly evident by the large reduction in the number of motor neurons in the SOD1^G93A^ astrocyte-containing cultures than normal astrocyte–neuron co-cultures (Figure 2k). Meso scale discovery (MSD) quantification revealed more than twofold reduction of NF levels in the SOD1^G93A^ astrocyte–neuron co-cultures (Figure 2l). Similar to growth factor withdrawal, many punctated structures on the axons occurred in the SOD1^G93A^ astrocyte co-cultures, but not in wild-type astrocyte co-cultures (Figure 2k). When 5D10 was added to the SOD1^G93A^ astrocyte–neuron cultures, there was about a twofold increase in motor neuron number and NF levels compared with control treatment, and a complete absence of axonal punctated structures (Figures 2k–m), thus demonstrating that 5D10 inhibits SOD1^G93A^ astrocyte-induced neurotoxicity.

**Figure 3:**
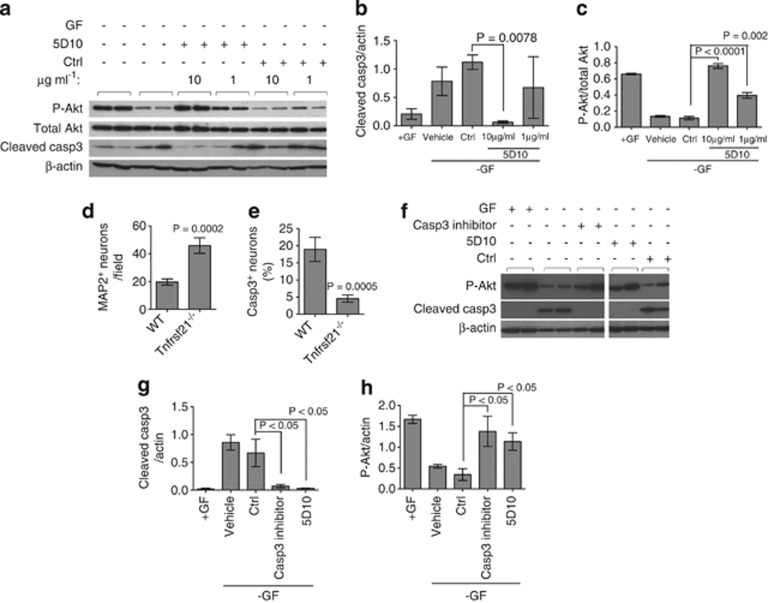
Anti-DR6 antibody promotes human motor neuron survival through inhibition of caspase 3 and activation of Akt phosphorylation. (**a**) Western blot analysis of cleaved casp3 and phosphorylated Akt in human motor neuron cultured in the presence or absence of growth factor and treated with 5D10 or control antibody (duplicate culture samples were analyzed). *β*-actin and total Akt were used as internal controls. Quantification of cleaved casp3 (**b**) and P-Akt (**c**) levels from **a** by densitometry. (**d**) Quantification of the number of surviving MAP2^+^ motor neurons purified from WT or *Tnfrsf21*^−/−^ mice following growth factor removal, *n*=15 fields/group. (**e**) Quantification of casp3^+^ motor neurons from **d**, *n*=15 fields/group. (**f**) Western blot analysis of cleaved casp3 and P-Akt in human motor neurons cultured in the presence or absence of growth factor and treated with or without casp3 inhibitor (duplicate culture samples were analyzed). *β*-actin was used as an internal control. Quantification of cleaved casp3 (**g**) and P-Akt (**h**) levels from **f** by densitometry. Data in **b**, **c**, **d**, **e**, **g**, and **h** were shown as mean±S.E.M. *P*-values were determined by two-tailed unpaired *t* test

To investigate the mechanism of action of the anti-DR6 antibody treatment survival effect on motor neurons, we quantified the levels of cleaved caspase 3 (casp3) and Akt phosphorylation (p-Akt) by western blot. Increased cleavage of casp3 is indicative of activating apoptotic death pathways, whereas increased p-Akt correlates with cell survival.^25, 35, 36^ Growth factor withdrawal led to a twofold increase of cleaved casp3 and a threefold decrease of p-Akt (Figures 3a–c). In contrast, 5D10 treatment decreased the levels of cleaved casp3, and led to a dose-dependent increase in the levels of p-Akt (Figures 3a–c). To further show that blocking DR6 reduces casp3 activation in motor neurons in the absence of growth factor, motor neurons from DR6-null (*Tnfrsf21*^*−/−*^) mice were evaluated. Similar to 5D10 treatment, when compared to wild-type motor neuron cultures, genetic deletion of DR6 led to a fourfold decrease of cleaved casp3 levels and more than fourfold increase in motor neuron survival determined by quantifying the microtubule-associated protein 2 (MAP2) (Figures 3d and e). To confirm that casp3 activation induces motor neuron death, we tested the casp3 inhibitor (Z-DEVD-FMK). As shown in Figures 3f and h, both 5D10 and Z-DEVD-FMK treatment led to decreased cleaved casp3 levels, and increased p-Akt levels. These data suggest that blocking DR6 promotes motor neuron survival by inhibiting casp3 activation and activating p-Akt survival signaling pathway.

### Blocking DR6 improves tissue integrity and motor function in SOD1 ^G93A^ mice

**Figure 4:**
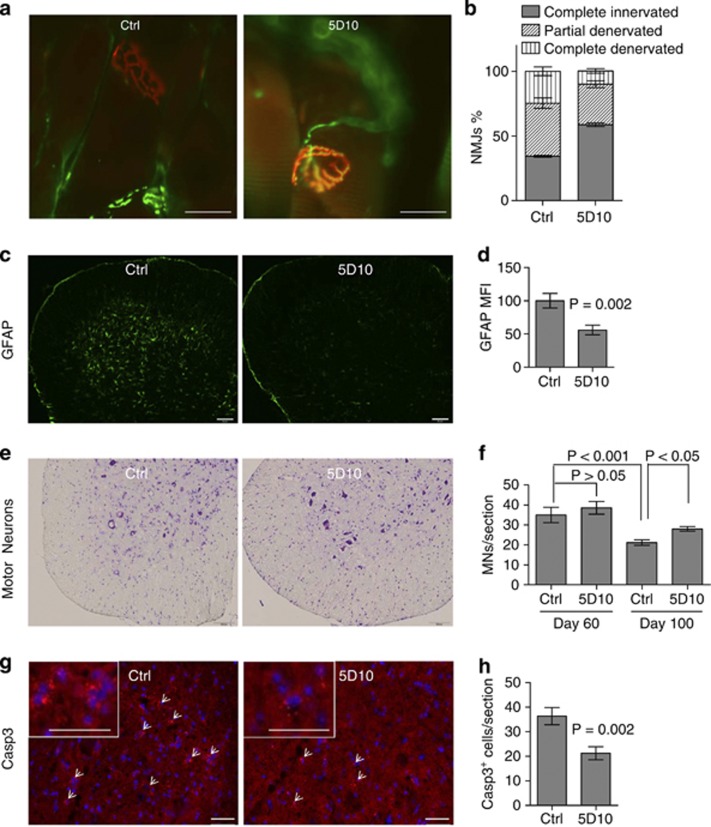
Blocking DR6 improves tissue integrity in SOD1^G93A^ mice. (**a**) IHC images of gastrocnemius muscle NMJs in 5D10 or control antibody treated SOD1^G93A^ mice (age 60 days), SV2 and NF (green), BuTx (red), scale bar=25 *μ*m. (**b**) Quantification of NMJs from **a**, *n*=∼300 NMJs/three animals/group. (**c**) IHC images of GFAP staining (green) in lumbar spinal cord of WT mice and 5D10 or control antibody treated SOD1^G93A^ mice (age 60 days), scale bar=95 *μ*m. (**d**) Quantification of GFAP staining from **c**, by mean fluorescence intensity (MFI) measurements. GFAP levels in control=100%, *n*=18 fields/3 animals/group. (**e**) IHC images of Nissl-stained lumbar spinal cord motor neurons in 5D10 or control antibody treated SOD1^G93A^ mice (age 100 days), scale bar=100 *μ*m. (**f**) Quantification of motor neuron number from **e**, ventral horn motor neurons (>20 *μ*m) were counted, *n*=9–18 sections/3–6 animals/group. (**g**) IHC images of cleaved casp3 staining (red) in lumbar spinal cord motor neurons of 5D10 or control antibody treated SOD1^G93A^ mice (age 100 days), arrows denote casp3^+^ cells, DAPI (blue), scale bar=50 *μ*m, inset scale bar=50 *μ*m. (**h**) Quantification of casp3^+^ cells in ventral horn region from **g**, *n*=9 sections/3 animals/group. Data in **b**, **d**, **f** and **h** were shown as mean±S.E.M. *P*-values in **b**, **d** and **h** were determined by two-tailed unpaired *t*-test, and in **f** were determined by one-way ANOVA followed by Bonferroni's test

**Figure 5:**
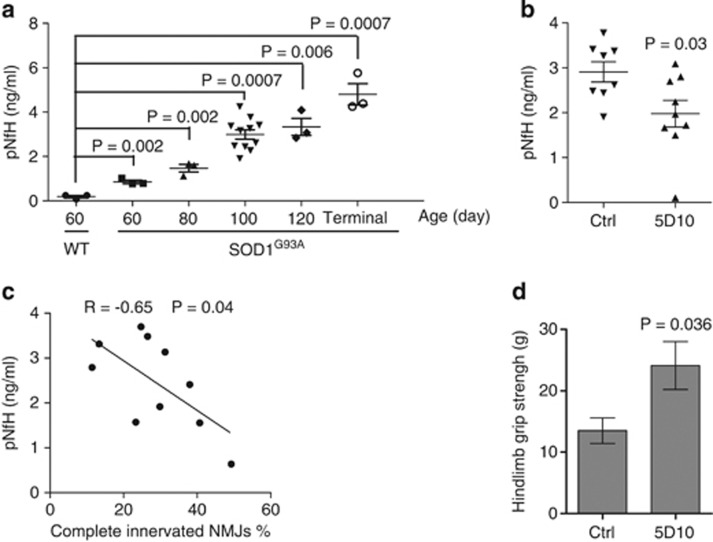
5D10 treatment deceases serum pNfH levels and improves hindlimb grip strength in SOD1^G93A^ mice. (**a**) Quantification of serum pNfH levels in WT or SOD1^G93A^ mice by MSD analysis, *n*=3–11 animals/group. (**b**) Quantification of serum pNfH levels of 5D10 or control antibody treated SOD1^G93A^ mice (age 100 days) by MSD analysis, *n*=8–9 animals/group. (**c**) Correlation analysis of serum pNfH levels and gastrocnemius muscle NMJs. (**d**) Hindlimb grip strength analysis (in grams) of 5D10 or control antibody treated SOD1^G93A^ mice (age 100 days), *n*=8–9 animals/group. Data in **a**, **b** and **d** were shown as mean±S.E.M. *P*-values in **a**, **b a**nd **d** were determined by two-tailed unpaired *t* test, and in **c** was determined by Pearson correlation analysis

The effect of blocking DR6 function on motor neuron survival *in vitro* prompted us to investigate if blocking DR6 had a neuronal protective effect on SOD1^G93A^ mice. As DR6 levels already had increased at the age of 60 days (Figure 1c), we tested if blocking DR6 had early beneficial effects at presymptomatic stage by protecting against NMJ denervation. Mice were treated intraperitoneally twice per week with 6 mg/kg 5D10 or isotype control antibody MOPC21, beginning at the age of 42 days to the end of the study. The antibody concentration and treatment regiments were selected on the basis of *in vitro* motor neuron survival data shown in Figure 2. The dosing regimen provides trough antibody concentrations in serum of >200 *μ*g/ml. Gastrocnemius muscle was dissected longitudinally and stained by IHC, with presynaptic and postsynaptic NMJs visualized by anti-SV2, anti-NF and *α*-bungarotoxin (BuTx) staining. NMJ classifications were divided in three categories: ‘completely innervated' (healthy functional NMJ with a complete overlap staining of SV2 staining with BuTx staining); ‘completely denervated' (no overlap staining) or ‘partially denervated' if there was partial overlap. For each animal, at least 100 NMJ were evaluated and the data are presented as percentage of each category. At the age of 60 days (before disease onset), there was extensive NMJ denervation of the gastrocnemius in control-treated SOD1^G93A^ mice, as 25% NMJs were completely denervated, 41% were partially denervated and 34% remained completely innervated (Figures 4a and b). In contrast, 5D10 treatment increased the percentage of completely innervated NMJ to 59% (10% NMJ were completely denervated, and 31% were partially denervated) (Figures 4a and b). There was no significant motor neuron death or sciatic nerve degeneration at the age of 60 days in SOD1^G93A^ mice as determined by Nissl staining of lumbar spinal cord section motor neurons and toluidine blue staining of the sciatic nerve, which is consistent with that of published literature (Figure 4f and Supplementary Figure 3). At the age of 60 days there was a significant decrease in astrocyte gliosis as indicated by less staining of GFAP in 5D10-treated SOD1^G93A^ animals compared with the GFAP staining in control-treated group (Figures 4c and d). At the age of 100 days, there was significant motor neuron loss in control-treated animal lumbar spinal cords, whereas the 5D10-treated group showed 33% more motor neurons, 35% more CC1^+^ oligodendrocytes, 49% increase in myelin basic protein (MBP) staining (Figures 4e and f, Supplementary Figure 4) and a 42% decrease in casp3^+^ cells (Figures 4g and h). As pNfH levels in serum are correlated with disease progression in SOD1^G93A^ mice^20^ (Figure 5a), we next tested serum pNfH levels in the 5D10 and isotype control-treated animals. As shown in Figure 5b, 5D10 treatment led to a twofold reduction of serum pNfH levels at the age of 100 days. Interestingly, the decrease in pNfH levels in serum had an inverse correlation with an increase in innervated NMJ numbers in 5D10-treated animals when compared with control-treated animals (Figure 5c). We next determined if the enhanced NMJ integrity and decreased serum pNfH levels correlated with motor function improvement as measured by a grip strength study. As shown in the Figure 5d, 5D10-treated animals showed a 79% increase in hindlimb grip strength when compared with control-treated littermates. This study strongly suggests that blocking DR6 has a neuronal protective effect on axon degeneration and functional improvement *in vivo*.

## Discussion

DR6 has emerged as an important regulator of oligodendrocyte^27^ and neuronal cell death.^25, 26^ Here we demonstrate that DR6 antagonism leads to motor neuron survival and axon protection *in vitro* and in SOD1^G93A^ mice. In cell culture, blocking DR6 with 5D10 promotes motor neuron survival and axon growth from a variety of insults, including growth factor withdrawal, reactive oxygen species induced by sodium arsenite and non-cell autonomous death induced by astrocytes expressing mutant SOD1 by blocking casp3 activation for cell death and promoting Akt activation for survival. In *in vivo* SOD1^G93A^ mice studies, treatment with anti-DR6 blocking antibody promotes motor neuron and oligodendrocyte survival, preserves NMJs and decreases astrocyte gliosis. The neuronal protection leads to improved motor function as measured by increased grip strength.

Glial cells have an important role in motor neuron death in culture. Others have reported that astrocytes from SOD1^G93A^ mice or sporadic human ALS patients regulate motor neuron cell death.^31, 32, 37, 38^ Here we demonstrate that blocking DR6 by 5D10 promotes motor neuron survival in motor neuron/astrocyte co-cultures containing astrocytes isolated from SOD1^G93A^ mice, providing evidence that DR6 contributes to this neurotoxicity. Consistent with this finding, anti-DR6 antibody treatment also led to decreased astrocyte gliosis in SOD1^G93A^ spinal cord *in vivo*.

Recently, several publications have demonstrated that oligodendrocytes have an important role in motor neuron survival by providing a metabolic support of axons.^28, 29, 30^ Our study also demonstrates that 5D10 treatment promotes oligodendrocyte survival in the spinal cord of SOD1^G93A^ mice. This suggests that blocking DR6 may have a dual role in motor neuron survival by blocking casp3 activation in motor neuron and by promoting the survival/maturation of oligodendrocytes that metabolically support axon integrity.

To determine anti-DR6 antibody's protective role in the SOD1^G93A^ animal model, mice were treated with 5D10 before disease onset. 5D10 treatment for as short as only 2 weeks protected NMJ integrity and decreased gliosis in the spinal cords, at a time when significant motor neuron loss had not yet occurred. The early protective effects on NMJ denervation and pNfH levels in serum could provide a quick screening approach for testing ALS therapeutics compared with the standard 5-month survival readout with SOD1^G93A^ mice.

Given the neuroprotective effect of 5D10 in SOD1^G93A^ mice, we further investigated whether 5D10 promotes SOD1^G93A^ animal survival and delays disease onset. SOD1^G93A^ mice were treated before disease onset. 5D10 treatment led to delayed disease onset and prolonged survival; however, inconsistent data were obtained in two independent studies (data not shown). The inability of our and other labs to generate consistent disease onset and survival data with this model^39^ suggests that these data may not be useful endpoints for assessing DR6, or other non-SOD1 mutation targets, to ALS. Furthermore, others and our studies suggest that the functional improvement, increased NMJ integrity and motor neuron survival do not necessarily correlate with mice survival.^40, 41, 42, 43^

p75^NTR^ regulates neuronal survival and death by binding to different ligands and signaling co-receptors.^44, 45, 46, 47^ p75^NTR^ promotes survival by binding tropomyosin-receptor-kinase receptor and enhancing its ability to bind and respond to neurotrophins.^45^ p75^NTR^ mediates cell death signal by complexing with sortilin upon binding to proneurotrophins.^46^ In addition, p75^NTR^ forms a tripartite complex with Nogo receptor 1 and Leucine rich repeat and Ig domain containing NogoR interacting protein1 to mediate axon growth inhibitory signals in response to Nogo, myelin-associated glycoprotein or oligodendrocyte myelin glycoprotein.^47^ p75^NTR^ has been implicated in motor neuron death in humans and in SOD1^G93A^ mice.^48, 49, 50^ p75^NTR^ expression is upregulated in spinal cord motor neurons,^48^ sciatic nerve^49^ and NMJs^50^ of SOD1^G93A^ mice and ALS post-mortem tissue.^48^ Therapeutically blocking or genetic depletion of p75^NTR^ in SOD1^G93A^ mice delayed disease onset and improved motor function and survival.^51, 52^ Recently, we discovered that DR6 and p75^NTR^ form a receptor complex that mediates cortical neuron apoptosis induced by A*β*.^26^ Disruption of this complex by using 5D10 to block the binding of DR6 to p75^NTR^ led to cortical neuron survival.^26^ As both DR6 and p75^NTR^ are upregulated in ALS and SOD1 mice, DR6 may induce motor neuron death through binding to p75^NTR^. This could be a direct result of increased expression of one or both components, or alternatively DR6 could interfere with neurotrophin-mediated survival effect of p75^NTR^. In fact, we found that neurotrophins compete for DR6 binding to p75^NTR^ (data not shown). In either situation, blocking the formation of the DR6/p75^NTR^ complex by 5D10 would contribute to motor neuron survival and neuronal protection.

ALS is a progressive and terminal motor neuron degenerative disease.^53^ The mechanisms leading to motor neuron death and axon degeneration are poorly understood. At least 15 genes have been identified that account for 30% of FALS cases.^54^ The majority of ALS cases are classified as sporadic with unknown etiology.^55^ Currently, there is no good treatment for ALS. Riluzole may have a marginal effect, prolonging lifespan by a few months.^56^ There is a significant need for therapeutics that protect NMJ integrity and lead to extended lifespan and functional improvement for ALS patients.

In summary, we demonstrate that DR6 hass an important role in regulating motor neuron survival and axon integrity, and that blocking its function with anti-DR6 antibody 5D10 promotes motor neuron survival *in vitro*. In the SOD1^G93A^ mouse model, blocking DR6 decreased gliosis and casp3 levels and increased the number of motor neurons and CC1^+^ oligodendrocytes in spinal cord, protected the integrity of NMJ in gastrocnemius muscle, led to motor function improvement and decreased pNfH levels in serum. Blocking DR6 function with an anti-DR6 antibody represents a potential therapeutic approach for treatment of ALS.

## Materials and Methods

### *In situ* hybridization

Fresh frozen spinal cords sections were obtained from SOD1^G93A^ mice (60 days of age) or age-matched control animals. As previously described,^27^ frozen sections were probed with digoxigenin-labeled DR6 antisense probe (5′-TAATACGACTCACTATAGGGGCTGGTGGGTAAGTTGTGGT-3′) and sense RNA probe (5′-ATTTAGGTGACACTATAGAACTCGCGGTACCTTCTCTGAC-3′). DR6^+^ motor neurons located in each ventral horn of spinal cords were counted.

### Western blot

Western blot were carried out as previously described^57^ using the 6A12 mouse anti-DR6 antibody (generated by Biogen Idec, Cambridge, MA, USA). Antibodies against cleaved casp3, phosphorylated and total Akt were from Cell signaling (Danvers, MA, USA) and rabbit antibody against β-actin were from Sigma (St. Louis, MO, USA). Band intensities were quantified by densitometry.

### Motor neuron survival

Rat motor neurons were isolated from E15–16 Sprague Dawley rat (Charles River, Wilmington, MA, USA) spinal cords using multiple discontinuous density gradients of NycoPrep.^58^ Mouse motor neurons were isolated from E13–14 WT C57BL/6 or *Tnfrsf21*^*−/−*^ mouse (Charles River). Embryonic stem cell-derived human motor neurons were purchased from California Stem Cell (Irvine, CA, USA). Neurons were plated in four-well chamber slides coated with poly-D lysine and laminin at the density of 3–5 × 10^4^ per well cultured in motor neuron culture media (Neurobasal/Dulbecco's Modified Eagle's medium/F12 medium supplemented with B27, 10 ng/ml human neurotrophin-3, 10 ng/ml human brain-derived neurotrophic factor, 10 ng/ml rat glial-derived neurotrophic factor and 25 ng/ml rat ciliary neurotrophic factor), as previously described.^58^ After 24 h incubation at 37 °C in humidified air with 5% CO_2_, neurons were treated with 0.5 mM sodium arsenite for 30 min. Cells were washed thrice with Neurobasal media and motor neuron culture media were added containing indicated concentration of anti-DR6 antibody 5D10 (5D10 generated as previously described^27^) or control antibody MOPC21. For growth factor removal study, cells were cultured in motor neuron culture media for 1–5 days, then media were replaced with 10 *μ*g/ml 5D10 or control antibody or 10 *μ*M Z-DEVD-FMK (Enzo life sciences, Farmingdale, NY, USA), but no growth factors. The cultures were continued to culture for additional 24 h, then harvested for western blot analysis, or fixed with 4% (*w*/*v*) paraformaldehyde for ICC study. Cells were stained with anti-NF (EMD Millipore, Billerica, MA, USA), anti-MAP2 (EMD Millipore), anti-βIII-tubulin (Covance, Denver, PA, USA) and anti-cleaved casp3 (Cell signaling) antibodies. Live motor neurons identified as NF^+^ or MAP2^+^ cells were counted. At least 12 randomly selected fields were counted at each treatment condition. Axon length was measured using Visiomorph software (Visiopharm, Broomfield, CO, USA).

### Astrocyte motor neuron co-culture

Mouse astrocytes were isolated from the brain of 2-month-old non-transgenic or SOD1^G93A^ transgenic mice. Briefly, mouse forebrain was dissected out, place in cold HBSS and chopped into ∼1-mm chunks with a sterile razor blade. DNAase and trypsin in HBSS were added to digest the tissue at 37 °C for 15 min. Cells were pelleted by centrifugation at 300 × *g*, suspended in DMEM plus 10% fetal bovine serum, and triturated with a glass pipette until homogeneous. The tissue was allowed to settle for 5 min, and then the suspension was passed through a 70-μm sieve (Falcon, Corning, NY, USA). The trituration step was repeated. The cell suspension was placed in cell culture flask (∼10^7^ cells per flask), and growed at 37 °C in humidified air with 5% CO_2_ until confluent. The cells were trypsinized and plated at the density of 5 × 10^4^/well in four-well chamber slides coated with poly-D lysine and laminin. After 24 h incubation at 37 °C and 5% CO_2_, purified rat motor neurons were added on top of astrocytes at the density of 5 × 10^4^/well, together with indicated concentration of 5D10 or control antibody. The cultures were grown for an additional 7 days, and then stained with anti-NF (EMD Millipore) and anti-GFAP (Dako, Carpinteria, CA, USA) antibodies as described above.

### Animal and therapeutic regiments

The transgenic SOD1^G93A^ mice used for these studies were the hybrid, high-copy strain (B6SJL-Tg (SOD1G93A) 1Gur/J, stock no. 002726) from the Jackson Laboratory (Bar Harbor, ME, USA). Mice were shipped at 6 weeks of age and maintained in an AAALAC-accredited research facility. All animal protocols were in accordance with US National Institutes of Health guidelines and approved by a local Institutional Animal Care and Use Committee. Mice were treated with 6 mg/kg anti-DR6 antibody or control antibody twice per week, given intraperitoneally in volumes of 10 ml/kg beginning at the age of 42 days to the end of the study, and tissues were harvested on days stated in the figure (3–6 animals/group). We generated SOD1^G93A^/*Tnfrsf21*^*−/−*^ and SOD1^G93A^/*Tnfrsf21*^*+/+*^ mice by crossing transgenic SOD1^G93A^ mice with *Tnfrsf21*^*−/−*^ mice.^27^

### Genotyping

Quantitative PCR was used to confirm SOD1^G93A^ transgene copy number relative to the endogenous gene IL-2. After excluding mice having very low copy number, all mice had 21±4 copies of the transgene. Genotype of *Tnfrsf21* locus was confirmed as previously described.^27^

### Immunohistochemistry

Freshly frozen human sporadic ALS post-mortem spinal cords tissues and non-ALS controls (age 37–82) were purchased from Tissue Solutions (Glasgow, UK) or bioBANC (Barcelona, Spain). DR6 staining was performed using the 6A12 mouse anti-DR6 antibody (Biogen Idec), together with VECTASTAIN ELITE ABC peroxidase and DAB substrate kit (Vector Labs, Burlingame, CA, USA). NeuN staining was performed using anti-NeuN antibody (EMD Millipore), together with VECTASTAIN ABC alkaline phosphatase and vector blue substrate kit (Vector Labs). Animal tissues were perfused with phosphate-buffered saline and post-fixed in 4% paraformaldehyde and then processed as previously described.^57^ For spinal cord, 10 *μ*m frozen sections were used for staining. Sections were stained with Crysel Violet (Nissl staining) or antibodies to NeuN (EMD Millipore), CC1 (EMD Millipore), MBP (Covance), GFAP (Dako), and cleaved casp3 (Cell signaling). Alexa488 or Alexa594 secondary antibodies were from Life Technologies (Grand Island, NY, USA). For quantification of motor neuron number, CC1^+^ oligodendrocyte number, casp3^+^ cell number and GFAP or MBP levels, at least three sections/animal, three animals/group were used. For gastrocnemius muscle neuromuscular junctions, 20-μm frozen sections were used for staining. Sections were stained with monoclonal antibody to SV2 (Iowa Developmental Hybridoma Bank, Iowa City, IA, USA), antibody to NF (Covance), and Alexa594 BuTx (Life Technologies). Secondary antibody was Alexa488-conjugated goat anti-mouse (Life Technologies). Images were collected using epifluorescence microscope (Leica, Buffalo Grove, IL, USA). Neuromuscular junctions were defined as ‘complete innervated' if there was complete overlap of the presynaptic marker (SV2, green) with acetylcholine receptor (AChR, red), revealed by BuTx staining, or ‘complete denervated' if there was no overlap or ‘partial denervated' if there was partially overlap. One hundred NMJs from each animal were evaluated and data are presented as percentage in each category. For quantification of NMJs, three animals/group were used. Toluidine blue staining of sciatic nerve to determine myelinated axons were described previously.^59^

### Grip strength

Grip strength was assessed using a grip strength meter (IITC Life Science, Woodland Hills, CA, USA). The animals were acclimatized on hindlimb T-bar for 3 consecutive days, at least 1 week before actual data collection. For hindlimb strength, a T-bar grip was used. The animal was held upright by the scruff of the neck and it was lowered over the pull bar with their back facing the meter. Once it grabs onto the bar, it is pulled backwards from the tail in a straight horizontal line while the animal's upright posture is maintained (to prevent it from grabbing onto the bar with its fore paw). When the animal lets go of the pull bar, it is placed back in the cage and the peak force is recorded from the display. The meter is tared (zero) and this procedure is repeated with the next subject. This was repeated 5 to 10 times within a 2-minute time-frame. The test was repeated in 2 consecutive days. The highest value was recorded for analysis.

### pNfH immunoassay

An electrochemiluminescence-based immunoassay was modified from a commercially available ELISA kit.^60^ Briefly, 96-well plates (Meso Scale Discovery, Rockville, MD, USA) were coated overnight with affinity-purified chicken antibody to pNfH (EnCor Biotechnology, Gainesville, FL, USA). All subsequent incubation steps were carried out for 1 h with constant shaking (400 r.p.m.) and were preceded by three wash steps with TBST buffer (10 mM Tris-HCl, 150 mM NaCl, 0.1% Tween-20, pH 7.5). Nonspecific binding was blocked with TBST buffer containing 3% bovine serum albumin (BSA). Sample and antibody dilutions were prepared with TBST buffer containing 1% BSA. The detection antibodies used were purified rabbit polyclonal antibody against pNfH (EnCor Biotechnology) and Sulfo-TAG-labeled goat anti-rabbit antibody (Meso Scale Discovery). Following a final wash, ECL read buffer (Meso Scale Discovery) was added and signal was measured using the MSD Sector Imager 6000 plate reader. A four-parameter weighted logistic fit curve was generated, sample concentrations were interpolated and analyzed using GraphPad Prism (GraphPad Software, Inc., La Jolla, CA, USA).

### Statistical analysis

GraphPad Prism software was used for statistical analysis. Comparison of mean values was conducted with unpaired Student's *t*-tests or one-way analysis of variance (ANOVA with Bonferroni post test). In all analyses, statistical significance was determined at the 5% level (*P*<0.05).

## Abbreviations

A*β*: *β*-amyloid
ALS: amyotrophic lateral sclerosis
BSA: bovine serum albumin
BuTx: *α*-bungarotoxin
casp3: caspase 3
DAPI: 4′ 6′-diamidino-2-phenylindole dihydrochloride
DR6: death receptor 6
FALS: familial ALS
GFAP: glial fibrillary acidic protein
ICC: immunocytochemistry
IHC: immunohistochemistry
MAP2: microtubule-associated protein 2
MBP: myelin basic protein
MSD: meso scale discovery
N-APP: N-terminal beta-amyloid precursor protein
NF: neurofilament
NMJ: neuromuscular junction
p75^NTR^: p75 neurotrophin receptor
p-Akt: Akt phosphorylation
pNfH: phosphorylated neurofilament heavy chain
PCR: polymerase chain reaction
SOD1: copper/zinc superoxide dismutase 1
SV2: synaptic vesicle protein 2
TBST buffer: 10 mM Tris-HCl, 150 mM NaCl, 0.1% Tween-20, pH 7.5
