# Supplementary figures and images for: Death receptor 6 (DR6) antagonist antibody is neuroprotective in the mouse SOD1G93A model of amyotrophic lateral sclerosis
Source: Cell Death Dis. 2013 Oct 10;4(10):e841–. doi: 10.1038/cddis.2013.378 (PMC3824687; doi:10.1038/cddis.2013.378)

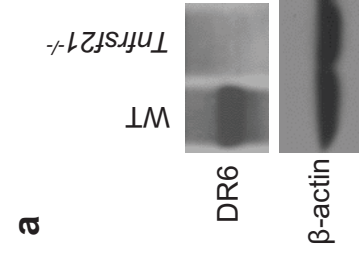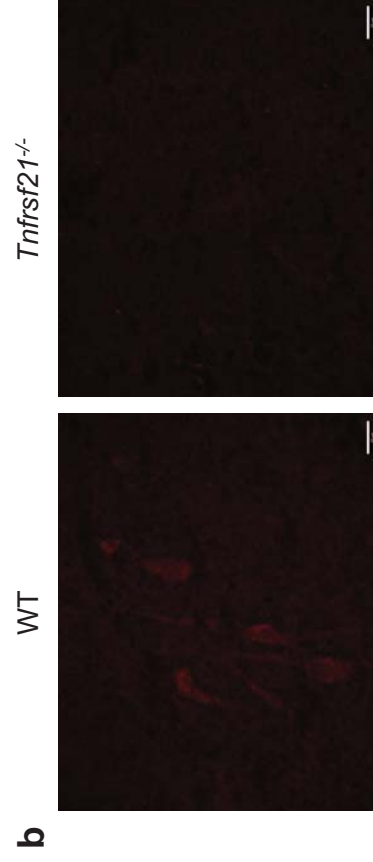

Supplementary figure 1

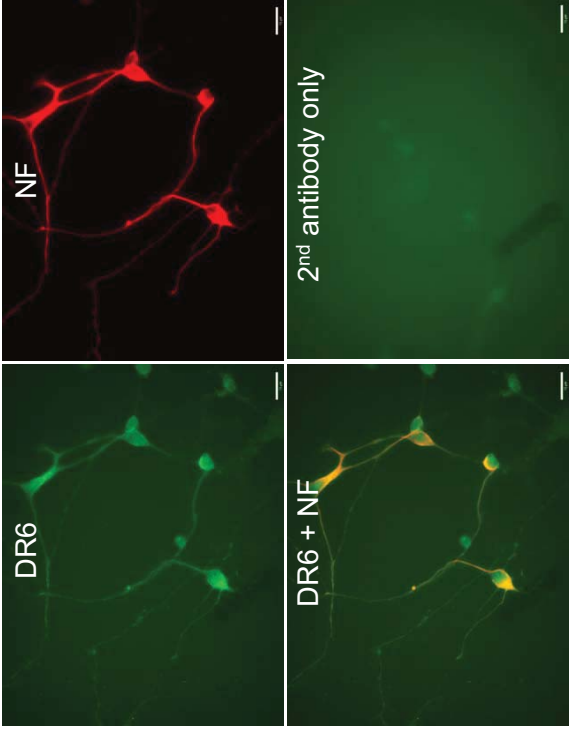

Supplementary figure 2

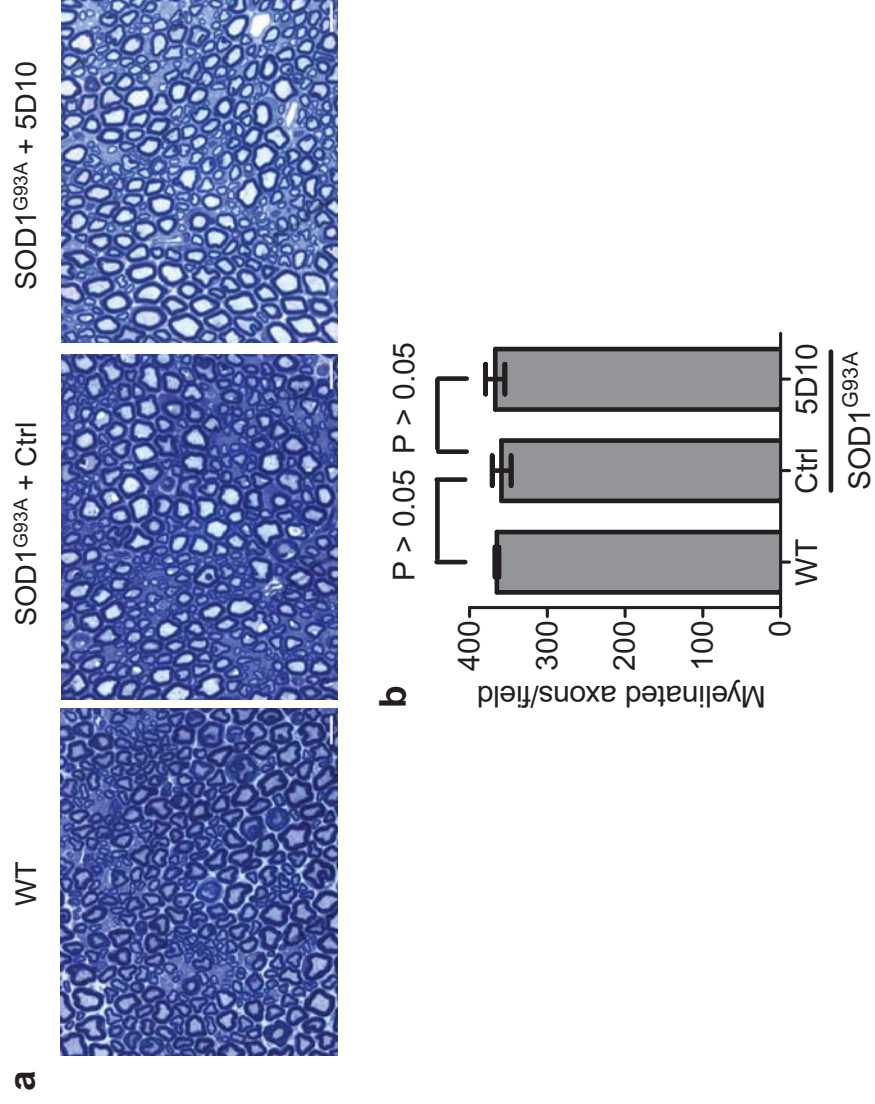

Supplementary figure 3

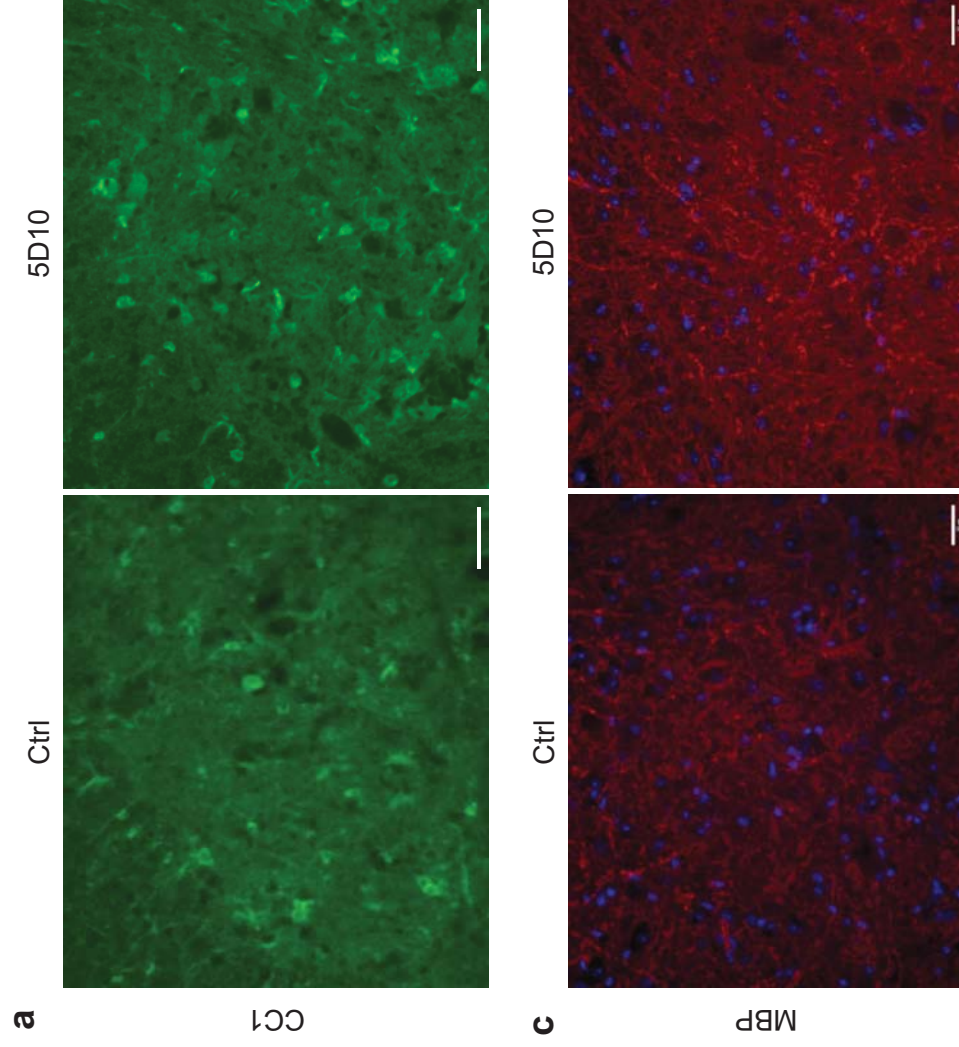

Supplementary figure 4

Supplement: Supplementary Figures [file cddis2013378x1.pdf]
