# Supplementary material for: Death receptor 6 (DR6) antagonist antibody is neuroprotective in the mouse SOD1G93A model of amyotrophic lateral sclerosis
Source: Cell Death Dis. 2013 Oct 10;4(10):e841–. doi: 10.1038/cddis.2013.378 (PMC3824687; doi:10.1038/cddis.2013.378)
Supplement: Supplementary Figures Legend [file cddis2013378x2.doc]

**Supplementary figure 1** Anti-DR6 antibody 6A12 specificity was determined by Western blot and IHC. (**a**) Western blot analysis of brain lysates of WT and *Tnfrsf21^-/-^* mice (age day 30) probed with 6A12. β-actin was used as an internal control. (**b**) IHC analysis of WT and *Tnfrsf21^-/-^* mouse spinal cord using 6A12, scale bar = 25 µm.

**Supplementary figure 2** ICC images of human motor neurons for DR6 expression. DR6 (green), NF (red), scale bar = 15 µm.

**Supplementary figure 3** No significant sciatic nerve degeneration at age day 60 in SOD1^G93A^ mice. (**a**) Toluidine blue staining to visualize sciatic nerve myelinated axons in WT mice and 5D10 or control antibody treated SOD1^G93A^ mice (age day 60), scale bar = 10 µm. (**b**) Quantification of sciatic nerve axons from **a**, n = 6 fields/3 animals/group.

**Supplementary figure 4** 5D10 treatment promotes oligodendrocyte survival in SOD1^G93A^ mice. (**a**) IHC analysis of CC1^+^ oligodendrocytes in lumbar spinal cord ventral gray matter of 5D10 or control antibody treated SOD1^G93A^ mice (age day 100), CC1 (green), scale bar = 50 µm. (**b**) Quantification of the density of CC1^+^ oligodendrocytes from **a**, n = 9 sections/3 animals/group. (**c**) IHC analysis of MBP staining of myelinated axons in lumbar spinal cord ventral gray matter of 5D10 or control antibody treated SOD1^G93A^ mice (age day 100), MBP (red), DAPI (blue),scale bar = 50 µm. (**d**) Quantification of MBP staining from **c**, by mean fluorescence intensity (MFI) measurements, MBP levels in control = 1, n = 9 sections/3 animals/group. Data in **b** and **d** were shown as mean ± s.e.m. P values were determined by two-tailed unpaired t test.
